# Context-sensitive smart glasses monitoring wear position and activity for therapy compliance—A proof of concept

**DOI:** 10.1371/journal.pone.0247389

**Published:** 2021-02-19

**Authors:** Kai Januschowski, Frank R. Ihmig, Timo Koch, Thomas Velten, Annekatrin Rickmann

**Affiliations:** 1 Eye Clinic Sulzbach, Knappschaft Hospital Saar, Sulzbach, Saarland, Germany; 2 Centre for Ophthalmology, University of Tuebingen, Tuebingen, Baden-Württemberg, Germany; 3 Fraunhofer Institute for Biomedical Engineering IBMT, Sulzbach, Saarland, Germany; Faculty of Medicine, Cairo University, EGYPT

## Abstract

**Purpose:**

To improve the acceptance and compliance of treatment of amblyopia, the aim of this study was to show that it is feasible to design an electronic frame for context-sensitive liquid crystal glasses, which can measure the state of wear position in a robust manner and detect distinct motion patterns for activity recognition.

**Methods:**

Different temple designs with integrated temperature and capacitive sensors were developed to realize the detection of the state of wear position to distinguish three states (correct position/wrong position/glasses taken off). The electronic glasses frame was further designed as a tool for accelerometer data acquisition, which was used for algorithm development for activity classification. For this purpose, training data of 20 voluntary healthy adult subjects (5 females, 15 males) were recorded and a 10-fold cross-validation was computed for classifier selection. In order to perform functional testing of the electronic glasses frame, a proof of concept study was performed in a small group of healthy adults. Four healthy adult subjects (2 females, 2 males) were included to wear the electronic glasses frame and to protocol their activities in their everyday life according to a defined test protocol. Individual and averaged results for the precision of the state of wear position detection and of the activity recognition were calculated.

**Results:**

Context-sensitive control algorithms were developed which detected the state of wear position and activity in a proof of concept. The pilot study revealed an average of 91.4% agreement of the detected states of wear position. The activity recognition match was 82.2% when applying an additional filter criterion. Removing the glasses was always detected 100% correctly.

**Conclusion:**

The principles investigated are suitable for detecting the glasses’ state of wear position and for recognizing the wearer´s activity in a smart glasses concept.

## Introduction

Amblyopia is one of the most common visual disorders in children [1]. It affects young patients in their daily life, influences job options and increases the risk for visual loss and trauma of the healthy eye throughout the children’s entire future [2–6]. This visual developmental disorder is best reversible in the sensitive phase of vision development [7]. Therefore, therapy should be started early. The current standard therapy of amblyopia starts with a refractive correction and usually continues with the occlusion of the better eye using a patch [8]. The success of the therapy also depends in particular on compliance and thus on actual wearing times of the occlusion patch. Because the better seeing eye is covered with an uncomfortable, often disfiguring adhesive plaster the acceptance of the therapeutic approach and adherence is often problematic [9].

Alternatively, electronic "shutter glasses" with liquid crystal lenses are known. They rhythmically occlude the better seeing eye by opacifying the lens [10,11]. However, compliance with these glasses also decreases during the course of therapy and is similar to patching over the course of three months [12]. Nevertheless, this approach is promising because most children need refractive correction and glasses could effectively replace occlusion patches for children with mild to moderate amblyopia using shuttering with combined liquid crystal lenses [13].

However, with the aim of improving acceptance and wear time compliance, a user-friendly interaction concept would have to be developed that goes beyond the current state of the technology and integrates sensor technology into the glasses to record wear and occlusion times as well as correct wear position. In addition, through adding sensory detection of movement-intensive activities during sports and games, the occlusion of the healthy eye could be sensibly suppressed in such accident-prone situations that require high visual responsiveness and spatial vision, so that the unrestricted vision of the healthy eye can be temporarily used.

It is the aim of this study to show in a proof of concept, that it is feasible to design an electronic frame for smart glasses that can measure wear time and occlusion time, measure the state of wear position in a robust manner and detect distinct motion patterns for activity recognition. We describe the development, functional testing and a proof of concept in a small group of healthy adults.

## Materials and methods

The following needs were defined for the development of context-sensitive liquid crystal glasses:

- Sensor technology for monitoring the state of wear position
- Sensor technology for activity dependent control of shutter function
- Acquisition and recording of raw sensor data
- Event data record of state of occlusion, wear position, and activity
- Memory capacity for event data record up to 3 months
- Compliance monitoring of defined wear time and occlusion time
- Generation of an optical feedback about the correct/wrong wear position and the daily adherence

### Electronic design of glasses frame

**Fig 1:**
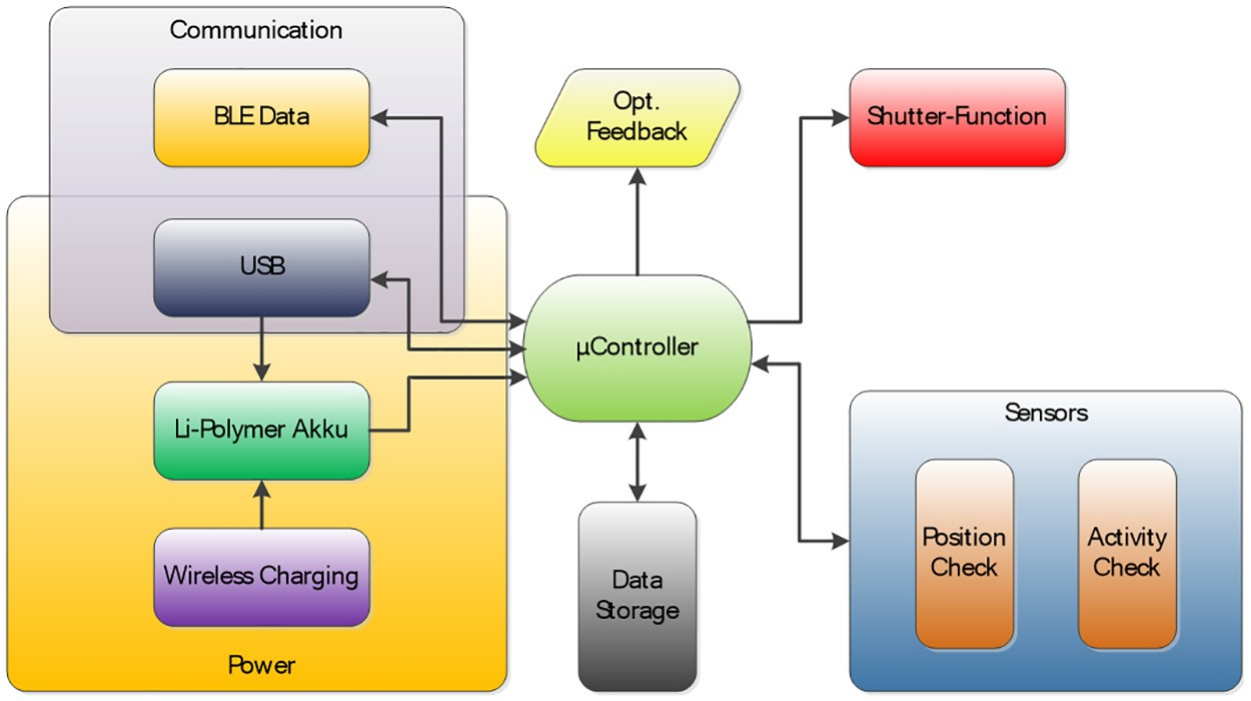
Block diagram of electronic design for glasses frame.

Fig 1 shows the block diagram with the electronic design of the glasses frame.

The functional blocks were implemented as follows:

Position Check—Sensors for monitoring the state of wear position

**Fig 2:**
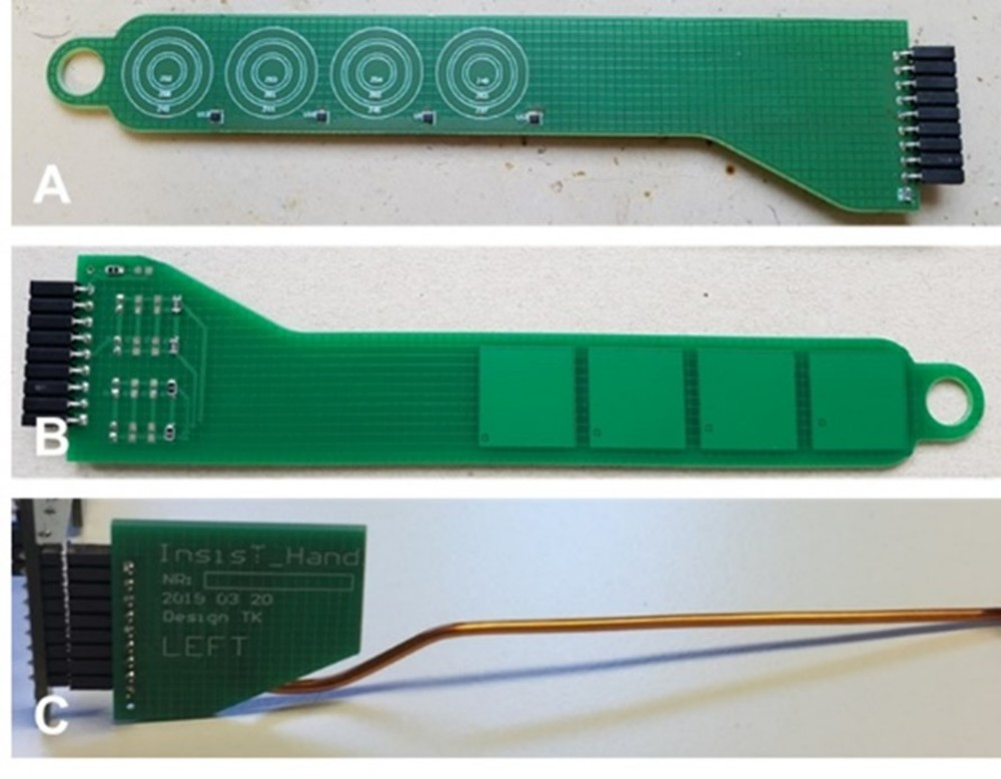
Photographs of electronic temple designs with capacitive and temperature sensors. (A) Initial temple design with four digital temperature sensors and four capacitive sensors with ring-shaped electrode structures with different diameters (5/10/15 mm). (B) Temple design with maximized electrode area using a rectangular shape. (C) Conductive metal temples made from round copper with lacquer coating (2 mm diameter), which could later be covered with a thin polymer layer.

Different temple designs were developed to realize the detection of the state of wear position (Fig 2).

The aim was to distinguish three states (correct position/wrong position/glasses taken off). In the initial temple design, a temperature sensor array consisting of nine miniaturized digital temperature sensors TMP112 (Texas Instruments, Dallas, TX, USA) with I²C interface, four on each temple and one on the nose bridge was realized. In addition, there is a body-contact sensor array consisting of nine capacitive sensors with ring-shaped electrode structures on the board with different diameters (5/10/15 mm), four on each temple and one on the nose bridge.

In a further design of the temples, we maximized the electrode area using a rectangular shape in expectation of improved sensing capability. As another option, we used conductive metal temples made from round copper with lacquer coating (2 mm diameter), which might later be covered with a thin polymer layer. The different temple designs were evaluated. For this purpose, the electronic glasses frame was used as a tool for sensor data acquisition. Six wrong wear positions were defined to check the sensitivity of the capacitive sensors; these positions were: Glasses are (1) too far down the nose, (2) at eyebrow level, (3) at forehead level, (4) on the head, (5) crooked with right temple raised, and (6) crooked with left temple raised.

#### Activity check—Sensors for activity dependent control of shutter function

To monitor the physical activity of the wearer, the 3-axis accelerometer LIS3DH (STMicroelectronics N.V., Amsterdam, Netherlands) with I²C interface was integrated.

#### Feedback mechanisms

LEDs are present as the feedback mechanism for the state of wear position, illuminated either green, yellow or red. Green—if the position is correct; yellow–if the glasses are taken off; red—if the position is wrong.

#### Communication

USB and Bluetooth 4.2 interfaces are present for communication between the electronic glasses frame and a PC or Smartphone/Tablet. In this study, USB communication was used to transfer recorded event data to a PC.

#### Power supply

USB and wireless charging are used as power supply for charging the battery of the electronic glasses frame. The receiving coil for wireless charging has a diameter of 10 mm. In this study, USB connection is used to charge the battery.

#### Control and processing unit

The 32-bit microcontroller STM32L476 (STMicroelectronics N.V., Amsterdam, Netherlands) with an ARM Cortex-M4 core is used as an energy-efficient and cost-effective control and processing unit. The flash memory S25FL127SABMFI101 (Cypress Semiconductor, San Jose, CA, USA) with a storage capacity of 128 Mbit (16 MB) is available as data memory.

**Fig 3:**
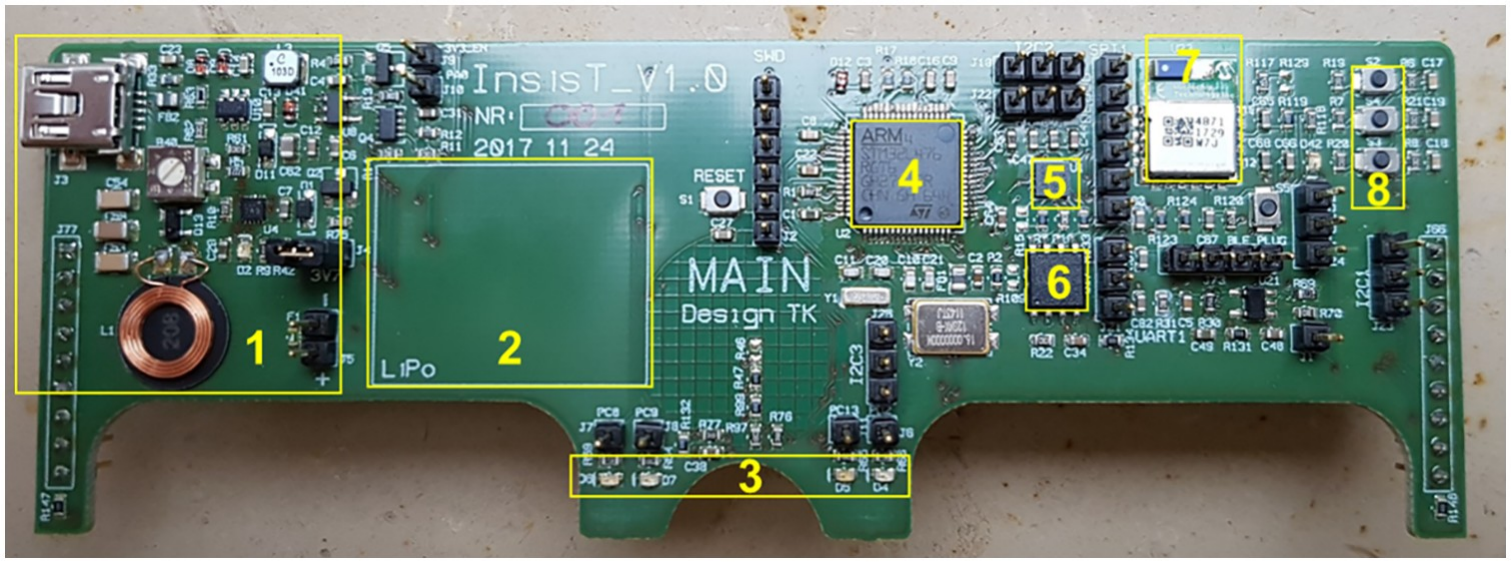
Photograph of electronic glasses frame. (1) Wireless charging and USB interface, (2) space for lithium-polymer battery, (3) feedback LEDs, (4) microcontroller, (5) accelerometer, (6) Flash memory, (7) Bluetooth low energy module, and (8) three push buttons.

The electronic glasses frame (Fig 3) is made from three pieces of FR4-based printed circuit board (Multi Leiterplatten GmbH, Brunnthal, Germany). The main part (130 mm x 45 mm) plugs into the temples (150 mm x 31 mm) and runs on a lithium-polymer rechargeable battery (3.7 V, 180 mAh). In this study, the electronic glasses frame is used as a data logger for event data acquisition in order to proof the principles of state of wear position detection and of activity recognition.

### Capacitive sensing principle for the state of wear position detection

**Fig 4:**
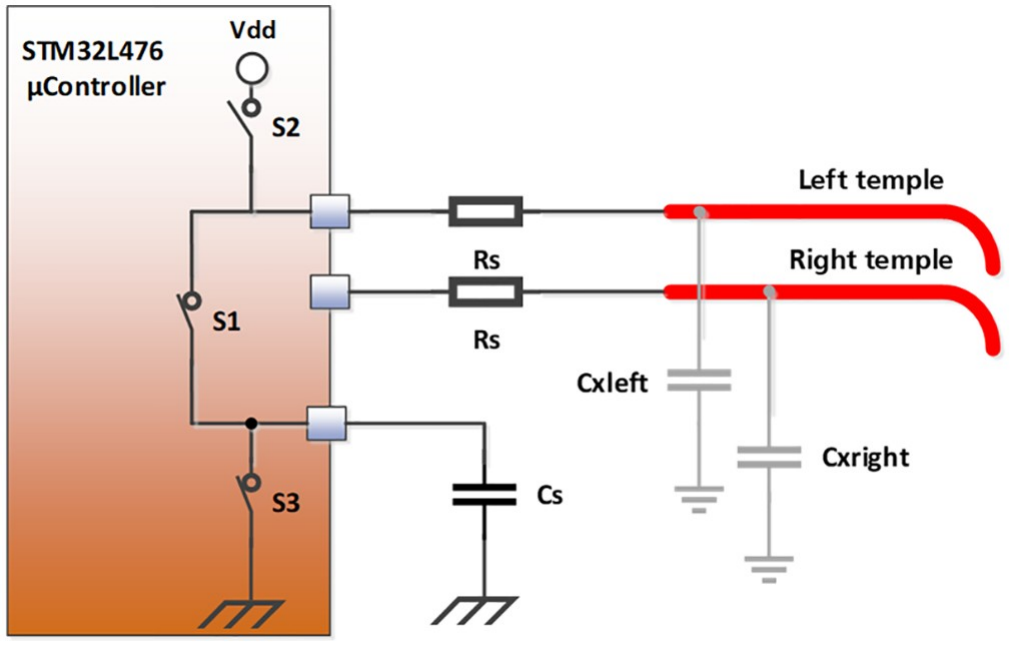
Capacitive sensing principle for the state of wear position detection.

We used the integrated touch sensing feature of the microcontroller, which is based on charge transfer, to develop the state of wear position detection. This touch sensing feature uses the surface charge transfer acquisition principle to measure the characteristic capacitance change for the three defined states of wear position. This acquisition principle consists in charging a sensor capacitance (Cx) and in transferring the accumulated charge into a sampling capacitor (Cs). The number of charge transfers required to reach the threshold voltage of Cs is a direct representation of the size of the electrode capacitance. Cs should have low tolerance and high temperature stability. The sensor capacitances Cxleft and Cxright are formed by the conductive temple elements (Fig 4).

With glasses taken off, the sensor capacitances to earth are very small so that many charge transfers are needed to reach the threshold voltage of Cs. These capacitances increase when the subject is touching the temples so that less charge transfers are needed. With glasses in correct position, these capacitances are represented by an individual count of charge transfers that can be compared to the subject’s measurement values recorded during calibration.

A charge transfer cycle is formed by six switching steps that are described in Table 1. The first step is a discharge of Cs. The second step is a dead time that lets all capacitors left floating. The third step charges the sensor capacitances followed by an additional dead time. Step five successively transfers the charge from Cxleft and Cxright to Cs. After the next dead time, step three to six are repeated until Cs reaches the logical „1" level. The number of charge transfer cycles to reach the logical „1" level at Cs is the measurement value that represents the size of the sensor capacitances.

**Table 1:** Switching steps of the capacitive sensing principle.

Step	S3	S2	S1	Description
1	Closed	Opened	Closed	Cs discharge
2	Opened	Opened	Opened	Dead time
3	Opened	Closed	Opened	Charge cycle (Cx charge)
4	Opened	Opened	Opened	Dead time
5	Opened	Opened	Closed	Transfer cycle (charge transferred to Cs)
6	Opened	Opened	Opened	Dead time

To reduce the influence of a change in ambient temperature on the capacitive sensing principle, multi-layer ceramic capacitors C1206C104F3GACTU (Kemet Corporation, Fort Lauderdale, FL, USA) with temperature-compensating C0G dielectric, were used for Cs in the measurement circuit. With regard to ambient temperature, the change in capacitance is negligible and is limited to ±30 ppm/°C at -55 °C to +125 °C.

### Activity recognition based on machine learning

The background to this effort is the sensory detection of movement-intensive activities that require high visual reactivity and spatial vision. Once the activity has been detected, this should then ideally suppress the occlusion of the healthy eye in a meaningful way so that the unrestricted vision of the healthy eye can be used temporarily.

For this part of the development, the electronic glasses frame was used as a tool for accelerometer data acquisition, which was necessary for algorithm development. In contrast to the detection of the state of wear position, the activity recognition is not based on an individual calibration but on a machine learning algorithm. Training data are necessary to teach such an algorithm for activity classification. For the recording of the training data, 20 voluntary healthy adult subjects were recruited at IBMT, 5 female and 15 male subjects. All subjects signed the informed consent for the experiment.

Each subject carried out the defined eight activities (lie, sit, stand, walk, stairs, run, jump and cycle) according to a defined protocol. Each activity was performed for 3 to 6 minutes per subject. When recording the data, special emphasis was put on enabling the subject to perform the activities individually. In total, about 775 minutes of accelerometer data with a measurement range of ±2 g were recorded.

**Fig 5:**
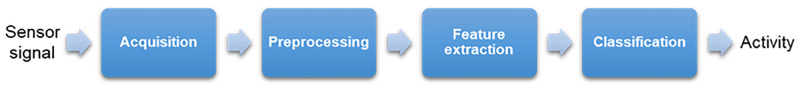
Workflow of activity recognition based on machine learning.

Fig 5 shows the workflow of activity recognition based on machine learning. After the accelerometer data acquisition, the recorded sensor data is pre-processed. This is necessary to apply a windowing of the recorded data. Windowing means that a time window is determined in which the recorded values are processed to detect any activity that has been carried out. When a certain activity is carried out, corresponding patterns occur within the sensor signals which can be used to assign the activity. Features are extracted within each window to describe these patterns. Then, each window is assigned to an activity using a machine learning algorithm (classification).

In this study, only features in the time domain and not in the frequency domain were extracted in order to keep the complexity low for the application on the microcontroller. According to our literature search [14–18], we selected 46 features and a window size of 2.56 seconds with a sample rate of 50 Hz and 50% overlap to begin the development of the activity classifier using MATLAB R2017b (The MathWorks Inc., Natick, MA, USA). To avoid redundant features, we performed a correlation analysis, after which 31 features were left. Then, a feature selection was carried out to further reduce the number of features to a minimum in order to reduce the model size while at the same time maintaining the classifiers’ accuracy as good as possible.

To choose a suitable classifier, 21 different models (2x decision trees, 2x discriminant analysis, 6x k-nearest neighbors (kNN), 6x support vector machines (SVM) and 5x ensemble classifiers) were compared with each other on the basis of a 10-fold cross-validation. This validation method was for example identified by Kohavi [19] as the best method for model selection in a comparative study of cross-validation and bootstrap. Besides a high classification accuracy, which is the proportion of correct predictions out of all predictions, we had the requirement that the classifier must be suitable to run on a microcontroller in an efficient way and in real time.

### Operation of the electronic glasses frame as data logger

The operation as data logger covers three modes: calibration, acquisition and data transfer via USB.

#### Calibration mode

When button 1 is pressed, the capacitive sensor values for the state "glasses taken off" are recorded. For this purpose, the glasses can lie on a table, for example, and must not be touched. The recording of the capacitive sensor values starts with a delay of 5 seconds. A fast flashing of the green LED indicates an active recording. After the green LED has stopped flashing, the first calibration step is finished. The next step is the recording of the capacitive sensor values for the state "correct position". For this purpose, button 2 must be pressed. The subject has 10 seconds to put on the glasses and to bring them into position before the actual recording takes place (yellow LED flashes).

#### Acquisition mode

The recording of event data can be started by pressing button 0. An active data acquisition is indicated by a LED flashing every second. Depending on the color of the LED, the detected state of wear position can be recognized: Red LED means “wrong position”, yellow LED means “glasses taken off", and green LED means “correct position”. During a running data acquisition, event data for activity and glasses position are logged with a time stamp. Data acquisition is stopped by pressing button 0 again.

#### USB mode

Logged data is transferred to a PC via USB connection using a terminal application.

### Tool for event data analysis

A tool for simplified decoding and analysis of the raw data was implemented in Excel. An event is characterized by decoded information such as date, time, consecutive event number and the states for wear position, occlusion and activity. The occlusion state is recorded as dummy data and has no effect in this study. An event is only recorded if one of the states for wear position, occlusion or activity changes. This leads to an efficient memory usage.

Further, there are columns for data analysis. These columns contain the states for activity and wear position recorded by the subject as well as the agreement (correct or error) with the recorded states of the electronic glasses frame. Also, there is a column with an additional filter criterion (referred to as the 3 windows filter) for evaluating the recorded activity and its agreement with the protocol. This filter criterion was expected to optimize the activity classification by changing the recorded activity state only if three successive classification results (windows) deliver the same state change.

### Proof-of-concept study

In order to test the above mentioned optimizations of the glasses, we carried out a proof of concept study at the end. The study procedure complied with the Declaration of Helsinki and was approved by the local Ethics Committee (Ärztekammer Saarland, 73/18). Informed and written consent was obtained from all healthy subjects after explanation of the nature and possible consequences of the study.

In total, four healthy adult subjects were included in the study (2 females, 2 males). The subjects were asked to wear the electronic glasses frame and to protocol their activities according to the defined test protocol. The test persons were asked to perform the following activities: sitting, standing, walking, running, jumping, cycling. This should be done with different wear positions of the glasses (correct position, front on the nose or on the head). This test protocol was not carried out by the subjects under controlled conditions in the laboratory, but in their everyday life. The state of wear position and the activity were varied repeatedly and several times in accordance with the protocol specifications. Each activity was performed continuously for at least 3 minutes, except for jumping for at least 1 minute, because it is exhausting. All time intervals and the corresponding states were recorded in writing by the subjects. We then evaluated the protocols and used the above mentioned Excel sheet for event data analysis. A blinded person evaluated the records and compared them with the protocols.

## Results

### Evaluation of temple designs

The investigation of different diameters of the body-contact sensors (5/10/15 mm) showed that the recorded capacitive sensor values are useful to differentiate the states of wear position (glasses put on or taken off) with all three sizes. As expected, the largest diameter provided the best sensitivity and resolution, since the difference in the recorded sensor values increased between the two states with increasing electrode area.

Six wrong wear positions were defined to check the sensitivity of the capacitive sensors. The results showed that it is possible to differentiate between correct and wrong wear positions for the diameters 10 and 15 mm. This could not be shown for the 5 mm diameter.

**Fig 6:**
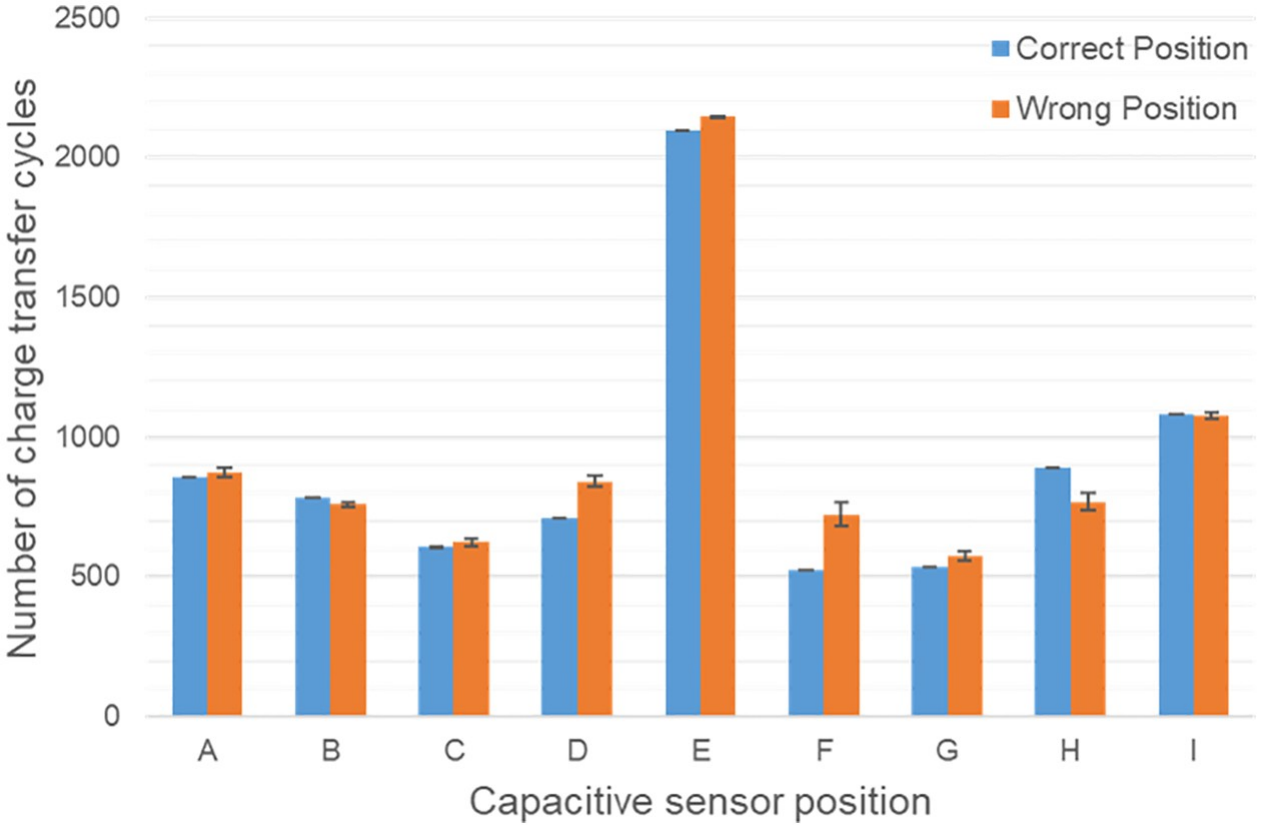
Number of charge transfer cycles of the nine body-contact sensors. (A to I with 10 mm diameter, n = 10) in case the glasses sit too far down the nose (blue bars) compared to the correct position (orange bars). In this case, recorded values of the sensors D/F/H provided a sufficient difference.

Fig 6 shows exemplary measurement results for the number of charge transfer cycles of the nine capacitive sensors (A to I with 10 mm diameter, n = 10) in case the glasses sit too far down the nose (orange bars) compared to the correct position (blue bars). In this case, the difference in the recorded values was the smallest compared to the other wrong positions. Nevertheless, there were at least three capacitive sensors (D/F/H) whose recorded values provided a sufficient difference. However, a deterioration of the sensor sensitivity depending on the hairstyle and head shape has been recognized. Therefore, despite individual calibration, the state of wear position may not be correctly detected with this temple design.

**Fig 7:**
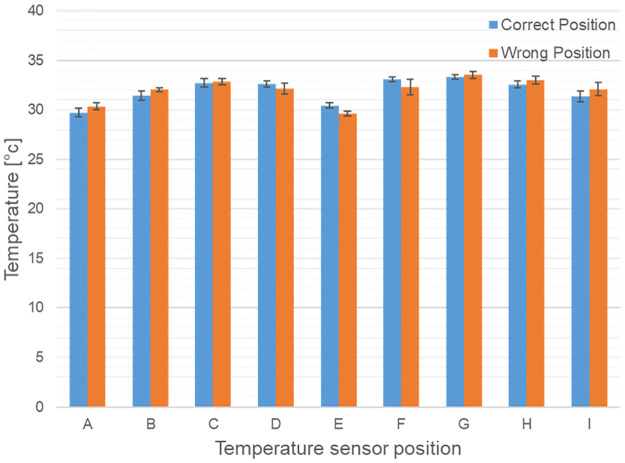
Temperature values of the nine temperature sensors. (A to I, n = 10) for the case that the glasses sit too far down the nose (blue bars) compared to the correct position (orange bars). The two states could not be distinguished.

The results of the nine miniaturized temperature sensors showed that due to their very slow behavior compared to the body-contact sensors, they were not suitable for providing quick feedback on the state of wear position. In addition, there was an "asymmetrical" response behavior, since cooling (taking the glasses off) takes considerably longer than warming (putting the glasses on). In addition, a strong influence of the ambient temperature was inherent. Fig 7 shows exemplary measurement results for the temperature values of the nine temperature sensors (A to I, n = 10) for the case that the goggles sit too far down the nose (blue bars) compared to the correct position (orange bars). The two states could not be distinguished. Thus, we decided not to use temperature sensors, thus reducing space requirements and costs of the hardware.

We could achieve a slight improvement of the sensing capability using the temple design with maximized rectangular electrode area. However, the deterioration of the sensor sensitivity depending on the hairstyle and head shape was still there because the rigid PCB material of the temples did not allow a sufficient fit that is as close as possible to the skin. We achieved further improvement of the sensing capability using the conductive metal temples because they provided sufficient electrode area and could be better adapted to the individual head shape and thus ensured close skin contact.

This design also has the advantage that the calibration routine is more efficient. We only need to record the values of two electrodes instead of nine. Tests have shown that the front electrode above the nose was also no longer needed to distinguish between different wearing states. These results prove that the capacitive sensing principle is suitable for this application.

### Validation of activity classifier

Highest accuracy values in all classes were achieved by the ensemble classifier Bagged Trees, the cubic SVM and the weighted kNN classifiers. However, it was noticeable in all of them that the differentiation between sit and stand classes was worse than in the other classes. Due to the positioning of the accelerometer at the forehead, this result is plausible. To achieve a higher classification accuracy, these two classes were combined into one class. This is acceptable, since these two activities are performed without moving the entire body and therefore a separation of these classes is not very relevant for the activity dependent control of the shutter function.

The highest accuracy was then achieved by the cubic SVM with 94.3%, followed by the Bagged Trees with 93.6% and the weighted kNN with 88.0%. Decisive for the selection of the classifier was the memory consumption of the respective model, which was highest for the Bagged Trees with almost 25.7 MB and lowest for the cubic SVM with 2.4 MB.

For further optimization, a feature selection was carried out for the cubic SVM in order to reduce the dimensionality of the feature matrix and at the same time the memory consumption to a minimum without worsening the classification accuracy too much. By selection of 13 features (see Table 2) and a variation of the error weight (increasing the error weight means that the number of support vectors should be reduced and thus also the size of the model) of the SVM, the memory consumption was reduced to about 983 kB while an accuracy of 93.4% was still achieved. By training this SVM with only two third of the recorded data, the memory consumption could be further reduced to about 722 kB while accuracy was only little less with 92,9%.

**Table 2:** Description of selected features for activity recognition.

Feature description	Formula
**mean(x), mean(y)**—average of an axis	$mean(a)=\frac{1}{n}\sum_{i=1}^{n}a_{i}$
**sd(x)**—standard deviation of an axis	$sd(a)=\sqrt{\frac{1}{n-1}\sum_{i=1}^{n}\left(a_{i}-{mean(a)}^{2}\right)}$
**meanAD(x)**—mean absolute deviation from mean value of an axis	*meanAD*(*a*) = *mean*(|*a* − *mean*(*a*)|)
**corr(x,y), corr(x,z)**—standardized linear relationship of two axes	${corr}_{a,b}=\frac{\frac{1}{n-1}\sum_{i=1}^{n}a_{i}\cdot b_{i}-(\frac{1}{n-1}\sum_{i=1}^{n}a_{i})\cdot (\frac{1}{n-1}\sum_{i=1}^{n}b_{i})}{\sqrt{(\frac{1}{n-1}\sum_{i=1}^{n}{\left(a_{i}-mean(a)\right)}^{2})\cdot (\frac{1}{n-1}\sum_{i=1}^{n}{\left(b_{i}-mean(b)\right)}^{2})}}$
**phi**—magnitude of the rotation angle of the accelerometer	$phi=\sum \frac{{AE}_{v}}{{AE}_{v}^{2}+{AE}_{h}^{2}}$
**magnitude**—magnitude of the total acceleration	$magnitude=\frac{1}{n}\sum_{i=1}^{n}\sqrt{\left(a_{x_{i}}^{2}+a_{y_{i}}^{2}+a_{z_{i}}^{2}\right)}$
**jerk(x)**—derivation of an axis according to time	$jerk=\sum_{i=1}^{n-1}\frac{a_{i+1}-a_{i}}{t_{i+1}-t_{i}}$
**minmax(y)**—difference between maximum and minimum of an axis	*minmax*(*a*) = *max*_abs_(*a*) − *min*_abs_(*a*)
**APF(x)**—average peak frequency of an axis	*APF(a)* = Σ*max*(*a*), *max*(*a*) > *minHeight*
**skewness(x)**—asymmetry of an axis compared to the normal distribution	$skewness(a)=\frac{\frac{1}{n}\sum_{i=1}^{n}{\left(a_{i}-mean(a)\right)}^{3}}{std{\left(a\right)}^{3}}$
**kurtosis(x)**—steepness/curvature of an axis compared to the normal distribution	$kurtosis(a)=\frac{\frac{1}{n}\sum_{i=1}^{n}{\left(a_{i}-mean(a)\right)}^{4}}{std{\left(a\right)}^{4}}$

**Fig 8:**
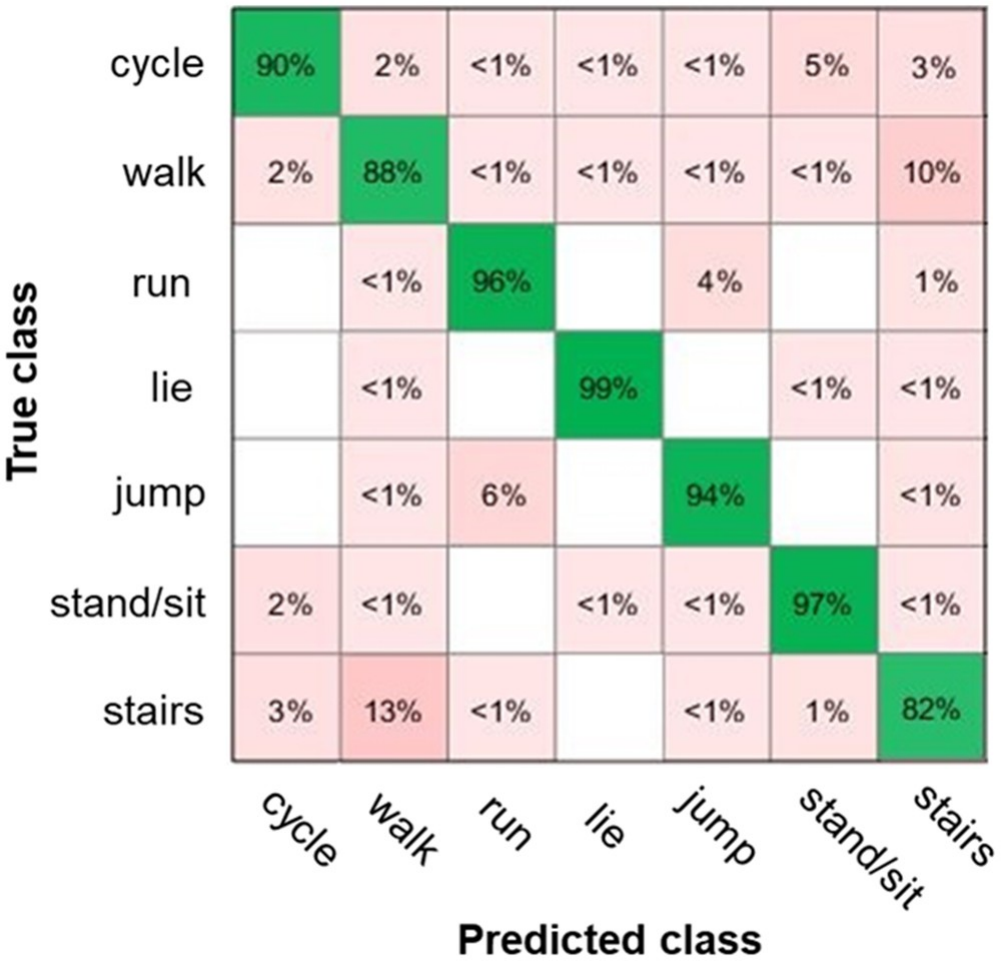
Confusion matrix for the cubic SVM classifier with 13 features.

The following Fig 8 shows the confusion matrix for this optimized cubic SVM classifier with an error weight of 6. Highest accuracy values of more than 95% were achieved for lie, stand/sit and run classes. Accuracy values of 94% and 90% were achieved for jump and for cycle classes, respectively. Lowest accuracy values of 88% and 82% were achieved for walk and for stairs classes, respectively. The differentiation between walk and stairs classes was worse than in the other classes. Finally, this classifier was ported to the microcontroller and integrated into its firmware.

### Precision of the state of wear position detection and of the activity recognition

Table 3 shows the individual results for the precision of the state of wear position detection and of the activity recognition. All subjects were well recognized if the glasses were in the wrong position. On the other hand, the correct position detection was only 47% for one of the subjects, which can be explained by an insufficient adaptation of the temples to the head shape. Removing the glasses was always detected 100% correctly. The individual activities were separated from each other to varying degrees. It was possible to distinguish very well between sitting, standing, lying, jumping and cycling. On the other hand, walking, running and going stairs were not distinguished well in some of the subjects.

**Table 3:** Individual results of the proof-of-concept study with four healthy adults.

Subject	Wear position correct [%]	Taken off [%]	Sit [%]	Lie [%]	Stand [%]	Walk [%]	Run [%]	Cycle [%]	Jump [%]	Stairs [%]
1	100	100	100	100	100	42,3	100	96,7	100	27,4
2	97,4	100	97,6	93,3	100	0	24,4	87,4	93,9	28,2
3	99,2	100	91	100	100	100	83,8	91,8	100	69,7
4	100	100	91,7	100	100	100	100	91	100	96,5

Table 4 shows the averaged results for the precision of the state of wear position detection and of the activity recognition. On average, there is a 91.4% agreement of the detected states of wear position. The activity recognition match is 82.2% when applying the 3 windows filter criterion and is thus 6% better than the unfiltered activity classification. Overall, these results are satisfactory, but at the same time they also show potential for improvement.

**Table 4:** Averaged results of the proof-of-concept study with four healthy adults.

Subject	State of wear position [%]	Activity [5] (3 windows filter)	Activity [%] (each window)
1	100	80,5	73,5
2	77,6	61,2	63
3	89,9	90,9	81,7
4	98,1	96	86,9
**Average**	**91,4**	**82,2**	**76,3**

## Discussion

This is the first study describing context-sensitive glasses with integrated sensors for monitoring the state of wear position as well as algorithms for activity dependent control of the shutter function. Finally, we were able to test the developed wearable device that proved reliable in a proof of concept study. The concept of our context-sensitive smart glasses will allow for the first time the continuous monitoring of the daily wear time and activity state. Our findings are important because they 1. potentially improve the current standard of therapy monitoring, 2. potentially improve patient adherence and therapeutic effectiveness.

1. Therapy monitoring: The success of occlusion therapy depends on the compliance that has been shown to be unsatisfactory [20–22]: Patients might wear the occlusion patch <50% of the recommended time [9]. By measuring the wear time and position of the glasses, and thus detecting true compliance, therapy schedules could be better adapted through feedback to parents and physicians. This is particularly important since there is a lack of evidence-based treatment of amblyopia.

The problem of objective measurement of compliance: temperature sensors on patches are reliable but are large and complicated to handle [23–26]. The lack of accuracy in temperatures higher than 33 °C [23,24] and no real position control are also a disadvantage [26]. This is in accordance with our results. We were able to show that the body-contact sensors integrated into the electronic glasses frame can safely and correctly detect the state of the glasses’ wear position and are even more reliable than the data from our temperature sensors. It would therefore be advisable to rely only on body-contact sensors reducing weight and power consumption of the electronic design. The fact that the conductive metal temples yielded reliable measurements makes an integration into smaller electronic frames possible.

2. Improving patient adherence and therapy effectiveness: The integration of sensors into the frame of the glasses and not into patches also has the advantage that any refractive correction can be compensated at the same time. Glasses could also be less stigmatizing than patches and studies have already shown that such occlusion therapy glasses are a promising alternative treatment for amblyopia in children [10,11,13]. In addition, the presented context-sensitive sensors in the glasses including algorithms for real-time evaluation could potentially detect the child’s activity and thus ideally adapt the occlusion to the situation, so that the unrestricted vision of the healthy eye can be temporarily used. In addition to safety, this also might increase the comfort and acceptance of the shutter glasses and thus the willingness of young patients to undergo therapy.

New perspectives: The integration of networked multimodal real-time sensor and actuator technology and computing capability in liquid crystal shutter glasses could provide feedback to both the patient and the parents if the glasses are worn or taken off incorrectly. In this study, we were able to show that the array of body-contact sensors placed in the temples and nose bridge of the glasses frame, together with a microcontroller algorithm, could successfully detect whether the glasses were correctly positioned. In addition, immediate feedback could be provided if the glasses were not correctly positioned or even if the glasses were removed. A correct position of the glasses is of crucial importance in therapy because it would theoretically be possible to "wear" the glasses at the tip of the nose and look over the glasses.

Based on this, context-sensitive control algorithms were developed which reliably detected the activity state in a proof of concept in adults. However, it should be mentioned that the accelerations and daily behavior differs significantly between adults and children. We consider the data of the protocols reliable and consider the measurements of activity and position monitoring robust. However, this is only a proof of concept study on a small number of subjects. In a next step a miniaturization and integration into a glasses frame for children is already planned.

## Conclusion

This is the first study showing that the principles studied for measuring the correct position and recognizing the wearer´s activity level are suitable and can be integrated into a concept of smart glasses.
